# Aberrant Spatial and Temporal Prefrontal Activation Patterns in Medication-Naïve Adults with ADHD

**DOI:** 10.3389/fpsyt.2017.00274

**Published:** 2017-12-05

**Authors:** Bun Yamagata, Yuichi Takei, Takashi Itahashi, Shenghong Pu, Jinichi Hirano, Masaru Mimura, Akira Iwanami

**Affiliations:** ^1^Department of Neuropsychiatry, School of Medicine, Keio University, Tokyo, Japan; ^2^Department of Neuropsychiatry, School of Medicine, Showa University, Tokyo, Japan; ^3^Department of Psychiatry and Neuroscience, Graduate School of Medicine, Gunma University, Gunma, Japan; ^4^Medical Institute of Developmental Disabilities Research, Showa University, Tokyo, Japan; ^5^Division of Neuropsychiatry, Department of Brain and Neuroscience, Faculty of Medicine, Tottori University, Tottori, Japan

**Keywords:** attention-deficit hyperactivity disorder (ADHD), near-infrared spectroscopy (NIRS), neuroimaging, cluster-based non-parametric randomization test, medication-naïve

## Abstract

Previous near-infrared spectroscopy (NIRS) studies using a verbal fluency task (VFT) have consistently reported that adults with attention-deficit hyperactivity disorder (ADHD) showed significantly smaller oxygenated-hemoglobin [oxy-Hb] activations in the prefrontal cortex (PFC) compared to those in healthy controls (HC). Despite this consistent evidence of brain dysfunction in ADHD, ADHD is currently diagnosed based only on subjective clinical and scoring measures, which are often unreliable. Hence, it is necessary to establish objective neuroimaging biomarkers for ADHD. While most NIRS studies have utilized averaged [oxy-Hb] values during the whole task period for group comparisons, we used a cluster-based non-parametric randomization test to compare the [oxy-Hb] time-course changes with a 0.1-s time resolution between drug-naïve adults with ADHD and HC, which may provide us with more details regarding abnormal prefrontal activation patterns in ADHD. A total of 101 participants, consisting of 63 drug-naïve adult individuals with ADHD and 38 HC, were included in this study. We identified that adults with ADHD showed significantly smaller [oxy-Hb] activations than those in HC at spatially and temporally connected clusters located in the bilateral PFC (more prominent on the left) and temporal brain region (more prominent on the left). We further found that aberrant [oxy-Hb] activation differs according to the time period during the task or according to brain location. Our findings indicate more detailed aberrant prefrontal and temporal activation patterns of ADHD compared with those in previous studies, possibly representing a biological marker for ADHD.

## Introduction

Although attention-deficit hyperactivity disorder (ADHD) has been classically recognized as a childhood disorder, previous longitudinal studies have found that in over half of all children with ADHD, symptoms persist into adulthood, especially with respect to inattention and impulsivity (1, 2). Thus, adults with ADHD are more likely to have serious problems in vocation, such as loss of employment, frequent job changes, instability or failure in important intimate relationships, and communication problems (2, 3). Furthermore, a recent cohort study has reported that 90% of adult ADHD cases lacked a history of childhood ADHD and suggests the possibility that adult ADHD may not be the same disorder as childhood ADHD (4). Therefore, the etiology for adults with ADHD may be different from those for children with ADHD.

A growing neuroimaging literature has addressed the neurobiological substrates of adults with ADHD using functional magnetic resonance imaging (fMRI). Deficits in neural circuits connecting brain regions of the prefrontal cortex and the striatum have been associated with this condition (5–7). Despite this consistent evidence of brain network dysfunction in ADHD, ADHD is currently diagnosed based only on subjective clinical and scoring measures, which are often unreliable. Hence, while it is necessary to establish objective neuroimaging biomarkers for ADHD, previous data for adults with ADHD have been limited.

Near-infrared spectroscopy (NIRS) is an optical neuroimaging technique that allows the non-invasive measurement of changes in the concentrations of oxygenated and deoxygenated hemoglobin ([oxy-Hb] and [deoxy-Hb], respectively), which is a reflection of changes in regional cerebral blood volume (8, 9). NIRS has the advantage of measuring hemodynamic changes at a higher temporal resolution (0.1-s time resolution) than fMRI. Previously, several studies using NIRS have revealed that children with ADHD showed abnormal prefrontal activations during executive function tasks including the trail-making test, Go/NoGo, and Stroop tasks (10–12). Furthermore, two NIRS studies using a verbal fluency task (VFT) reported that adults with ADHD showed significantly smaller [oxy-Hb] activations during the VFT relative to healthy controls (HC) in the prefrontal cortex (PFC) (13, 14).

In the statistical analysis of NIRS data, the multiple comparisons problem (MCP) is a concern. NIRS data have a spatiotemporal structure that includes 52 channels covering the bilateral frontotemporal brain region and 1,251 time-points throughout the whole experimental period (total duration of 125.1 s with 0.1-s time resolution). This number is usually on the order of several tens of thousands of data points. In most neuroimaging studies, including functional and structural MRI, the use of the family-wise error rate (FWER) is one way to solve the MCP by lowering the critical alpha level; however, this approach is overly conservative for NIRS because of the large number of dependencies in space and time. Thus, in most NIRS studies including two-adult ADHD studies (13, 14), two-dimensional data are reduced to one-dimensional data by averaging the [oxy-Hb] values over either a selected set of time-points or a selected set of channels. Using this approach, however, it is possible that fine group differences in the time-course or brain location of [oxy-Hb] activations have not been adequately assessed in adults with ADHD.

Contrary to the familiar parametric statistical framework like the FWER, a cluster-based non-parametric randomization test is an alternative method that combines cluster-based inference with assumption-free techniques like permutation, which have been shown to outperform analytic techniques in solving the MCP (15). In particular, this technique allows comparisons of the [oxy-Hb] values for each channel and the time-point between the two conditions by taking into account spatial and temporal adjacency: only clusters over a given significance level are kept. Therefore, this method has been widely used in electroencephalography (EEG) and magnetoencephalography (MEG) studies because of their spatiotemporal structures.

In this study, we used the cluster-based non-parametric randomization test to compare [oxy-Hb] time-course alterations with a 0.1-s time resolution between drug-naïve adults with ADHD and HC, which may provide more detailed prefrontal activation patterns in ADHD. We hypothesized that during the VFT, adults with ADHD would show significantly smaller [oxy-Hb] activations, compared with HC, in prefrontal and temporal brain regions.

## Materials and Methods

### Participants

A total of 101 participants, consisting of 63 drug-naïve adult individuals with ADHD and 38 HC, were included in this study (Table 1). Participants with ADHD were recruited from Showa University Hospital (Tokyo, Japan). HCs were recruited from the community through website advertisements at Showa University Hospital (Tokyo, Japan) and Tottori University Hospital (Tottori, Japan). All the individuals with ADHD were diagnosed by experienced psychiatrists (Bun Yamagata and Akira Iwanami) based on Conners’ Adult ADHD Diagnostic Interview for DSM-IV (16). Participants were included if they had no lifetime history of bipolar disorder, psychosis, obsessive-compulsive disorder, drug or alcohol misuse, or neurological disorder. Furthermore, none of the participants reported an unstable medical condition or a history of significant head trauma. To rule out any psychiatric conditions, experienced psychiatrists (Bun Yamagata and Akira Iwanami) examined all the participants using the Mini-International Neuropsychiatric Interview (17, 18). The intelligence quotient (IQ) scores of all the ADHD participants were evaluated using a 25-item short version of the Japanese Adult Reading Test (JART25) (19, 20), which is composed of 25 Japanese irregular words as an estimate of IQ. The Conners’ Adult ADHD Rating Scale (CAARS) (21) was used to assess the severity of inattention and hyperactivity symptoms. To assess the presence and severity of autistic traits in ADHD participants, the Autism-Spectrum Quotient (AQ) was administered (22). We used the Japanese version of the AQ, which has been assessed for its internal consistency reliability, test-retest reliability, and discriminant validity (23). After an extensive description of the study, written informed consent was obtained from all the study participants. The study protocol was approved by the Ethics Committee of Showa University and Tottori University and was prepared in accordance with the ethical standards of the Declaration of Helsinki.

**Table 1:** Demographic and clinical characteristics.

	ADHD	HC	*P* value
*n*	63	38	
Age (years)	30.9 ± 7.8	29.8 ± 4.5	0.41
Sex (female/male)	27/36	10/28	0.1
Education (years)	15.5 ± 2.3		
Estimated-IQ	109.5 ± 9.1		
AQ total score	31.0 ± 7.8		
CAARS inattention	17.5 ± 5.3		
CAARS hyperactivity	8.3 ± 5.5		
Task performance	14.7 ± 4.6	14.9 ± 4.5	0.83

*ADHD, attention-deficit hyperactivity disorder; HC, healthy control; IQ, intelligence quotient; AQ, autism-spectrum quotient; CAARS, Conners’ Adult ADHD Rating Scale*.

### Verbal Fluency Task

The task procedure in the present study was similar to that used by Takizawa et al. (24). Participants sat in a comfortable chair and were instructed to relax and to avoid any major body movements, so as to avoid artifacts. The [oxy-Hb] changes were measured during the VFT (letter version), which was comprised of a 30-s pre-task baseline, 60-s VFT, and a 60-s post-task baseline. For the pre- and post-task baseline periods, the participants were instructed to consecutively repeat the five Japanese vowels (“a,” “i,” “u,” “e,” “o”) aloud.

During the task period, participants were instructed to produce as many nouns as possible beginning with a designated syllable without the use of repetitions or proper nouns. Three sets of initial syllables (A;/to/,/se/,/o/, B; /a/,/ki/,/ha/, C; /na/,/i/,/ta/) were presented in counterbalanced order to the subjects while changing each syllable every 20-s during the 60-s task. The subtraction method (task minus pre- and post-task baselines) minimized the vocalization effects during the VFT. The total number of correct words generated during the VFT was taken as a measure of task performance.

### NIRS Measurements

We utilized a 52-channel NIRS machine (ETG-4000 Optical Topography System; Hitachi Medical Co., Japan) using two different wavelengths (695 and 830 nm) with a 0.1-s time resolution to measure the relative changes in absorbed near-infrared light. These changes were transformed into concentration changes of [oxy-Hb], [deoxy-Hb], and total-hemoglobin [total-Hb; sum of oxy-Hb and deoxy-Hb] as indicators for brain activity using a modified Beer–Lambert law (25). The unit was mM × mm (i.e., the changes in the chromophore concentration depend on the path length of the near-infrared light).

We utilized 33 optrodes consisting of 17 light emitters and 16 detectors with an inter-optrode distance of 30 mm. A channel (measuring point of activation) was defined as the region between one emitter and one detector. Thus, the optrode set consisted of 52 channels and measures [Hb] in the bilateral prefrontal [approximately dorsolateral (Brodmann’s area (BA) 9, 46), ventrolateral (BA 44, 45, 47), and frontopolar (BA 10)], superior temporal, and middle temporal cortical regions. The panel was fastened to the head by elastic straps. The correspondence between the optrode positions and the measurement areas on the cerebral cortex was confirmed based on a previous multi-subject study of anatomical craniocerebral correction *via* the international 10–20 system (26, 27).

The obtained data were analyzed using the “integral mode”; the pre-task baseline was determined as the mean over a 10-s period just prior to the task initiation, and the post-task baseline was determined as the mean over the last 5 s of the post-task period; a linear fitting was applied to the data between these two baselines. A moving average using a window width of 5 s was applied to remove any short-term motion artifacts. Furthermore, we calculated the median and the SD of each group at each channel and rejected data above or below three SDs of the median value. As a result, we rejected 28/3,276 (total subjects × total channels) channel time series from the ADHD group and 20/1,976 data points from the HC group. We focused on the [oxy-Hb] concentrations during the whole experimental period including the pre- and post-task baselines and the task period, since the [oxy-Hb] change was assumed to reflect cognitive activation more directly than the [deoxy-Hb] change as shown by a stronger correlation with the blood-oxygenation level-dependent (BOLD) signal measured using fMRI (28).

### Data Analysis

Regarding the clinical and behavioral data, we compared the age, sex ratio, and total numbers of produced words in the VFT between the groups using the Student’s *t*-test and the chi-squared test. To evaluate the condition difference (ADHD vs. HC) in the [oxy-Hb] data, we first compared the time-course [oxy-Hb] changes during the whole baseline and the VFT periods using a cluster-based non-parametric randomization test (*P* < 0.05 corrected) based on Monte-Carlo estimates, as described by Maris and Oostenveld (29). This analysis effectively controls the Type I-error rate with respect to multiple comparisons over channels and time-points, because NIRS has 52 channels and there are 1,251 time-points during the whole experimental period including a 10-s pre-VFT, 60-s VFT, and 55.1-s post-VFT (total duration of 125.1 s with a 0.1-s time resolution). This is achieved by clustering neighboring channels and time-points. For first-level statistics, the channels and time-points over a threshold (*F* = 14.9; calculated from *P* = 5e-14 using the probability density function of the subjects distribution) were identified from a one-way analysis of variance (ANOVA). Subsequently, spatially and temporally contiguous points in terms of channels and time-points over this threshold were defined as a cluster. Then, the sum of *F* values for a given cluster was used for cluster-level statistics. By repeatedly resampling the data across all the subjects, cluster-level *F* values were created from 10,000 randomization routines, and a histogram of the largest summed *F* value distribution (H0 distribution) was constructed. For the second-level statistics, we first performed an ANOVA in the original data set and obtained the sum of the *F* values in each spatially and temporally contiguous cluster. The *P* value was estimated from that summed *F* value of the original dataset according to the proportion of the randomization distribution (H0 distribution) (detailed in Figure 2). We used a “spatio_temporal_cluster_test” in MNE-python (30) for the cluster-based non-parametric randomization test of the NIRS data.

We also performed a correlation analysis between clinical measurements including AQ, CAARS, and VFT and the mean [oxy-Hb] values that were extracted from each cluster showing a significant group difference in the cluster-based non-parametric randomization test.

## Results

### Demographic Characteristics

There were no significant differences in age, sex ratio, and VFT performance between the HC and ADHD groups (Table 1).

### Group Comparison in Cluster-Based Non-parametric Randomization Test

**Figure 1:**
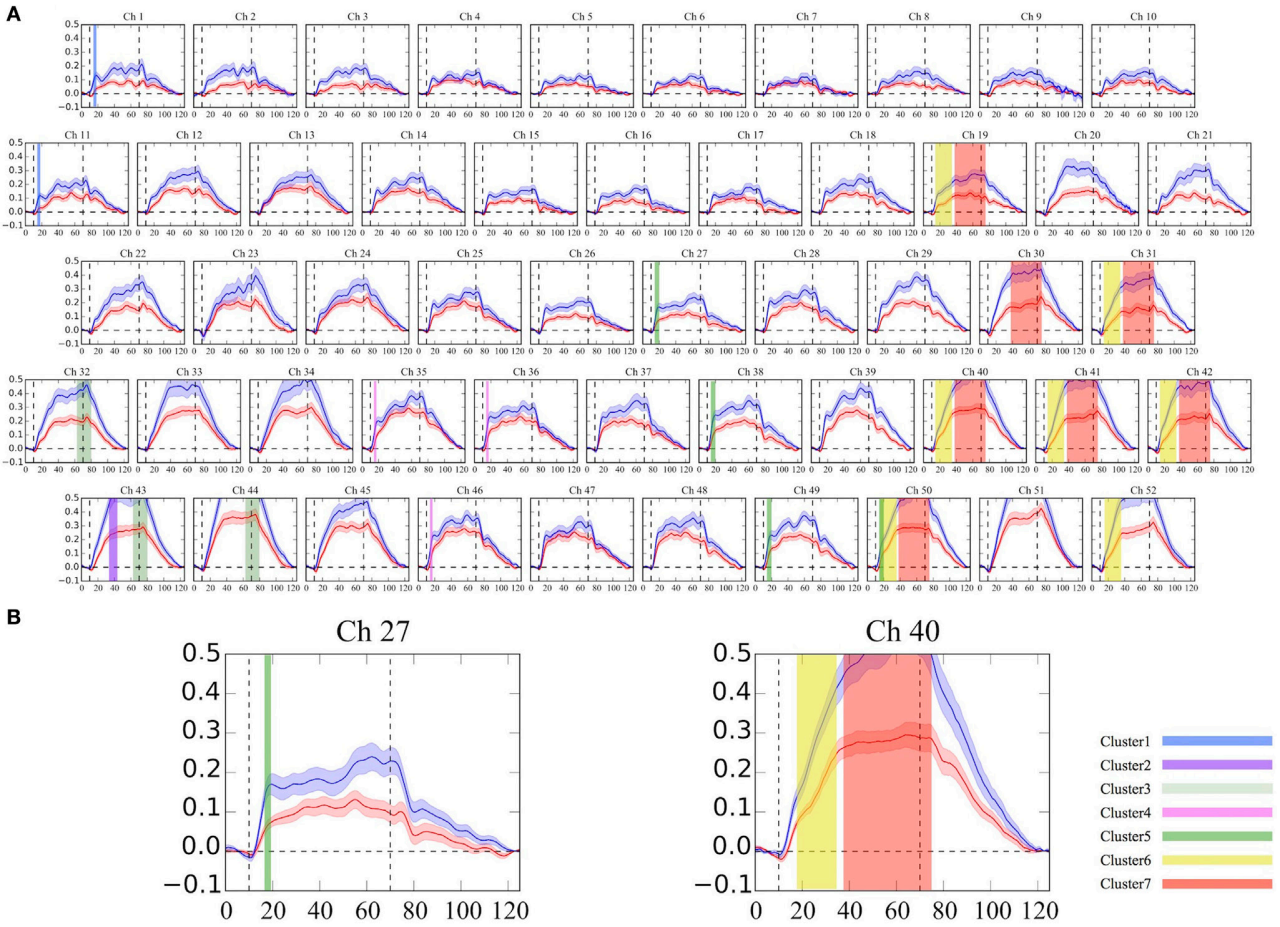
Grand average waveforms of [oxy-Hb] changes during the VFT in the frontotemporal brain regions. **(A)** The 52 measuring positions are labeled as Ch1–Ch52, from the right temporal to the left temporal regions. **(B)** Example of detailed time-course [oxy-Hb] changes with a 0.1-s time resolution between the groups at Ch27 and Ch40. The red line represents the ADHD group, and the blue line represents the HC group. The same colored clusters represent spatially and temporally contiguous points with significantly smaller [oxy-Hb] activations during the VFT in the ADHD group, compared with the HC group (*P* < 0.05 corrected). Each cluster number corresponds to Table 2. ADHD, attention-deficit hyperactivity disorder; HC, healthy controls; VFT, verbal fluency task; [oxy-Hb], oxygenated-hemoglobin.

As was suggested by a visual inspection of the overall NIRS images, the [oxy-Hb] values activated by the VFT were significantly smaller in the adult participants with ADHD than in the HC at seven clusters located in the bilateral prefrontal and temporal brain regions (Table 2; Figure 1). Interestingly, we found that the group difference of [oxy-Hb] activations varied according to brain location and time period during the task. In particular, a group difference in [oxy-Hb] values in the bilateral frontal pole (more prominent on the left) was observed only for a short time immediately after the activation task had started. In contrast, group differences were seen during almost the whole task period at several clusters located in the left DLPFC, the ventrolateral prefrontal cortex (VLPFC), and the superior temporal gyrus.

**Table 2:** Brain regions showing significantly smaller [oxy-Hb] activations during the VFT in ADHD compared to HC.

Cluster	Brain region	Channel	Time-points (duration time)	Summed *F* value	*P* value
1	R-supramarginal gyrus	1, 11	14.20–18.10 (3.90)	1,268.6	0.016
2	R-middle temporal gyrus	43	33.30–43.70 (10.40)	1,772.7	0.011
3	R-superior temporal gyrus, R-middle temporal gyrus	32, 43, 44	62.90–79.60 (16.70)	6,094.3	0.002
4	R-middle frontal gyrus, R-inferior frontal gyrus	35, 36, 46	14.10–17.40 (3.30)	1,013.8	0.022
5	L-superior frontal gyrus, L-middle frontal gyrus, L-inferior frontal gyrus	27, 38, 49, 50	13.80–19.60 (5.80)	2,597.2	0.006
6	L-middle frontal gyrus, L-inferior frontal gyrus, L-precentral gyrus, L-postcentral gyrus, L-superior temporal gyrus, L-middle temporal gyrus	19, 30, 31, 40, 41, 42, 50, 52	15.50–35.90 (20.40)	15,685.8	<0.001
7	L-middle frontal gyrus, L-inferior frontal gyrus, L-precentral gyrus, L-postcentral gyrus, L-superior temporal gyrus, L-middle temporal gyrus	19, 21, 30, 31, 40, 41, 42, 50	37.20–75.10 (37.90)	28,429.2	< 0.001

*L, left; R, right; ADHD, attention-deficit hyperactivity disorder; HCs, healthy controls; VFT, verbal fluency task; [oxy-Hb], oxygenated-hemoglobin*.

### Correlation Analysis

There were no correlations between the mean [oxy-Hb] values extracted from each of the seven clusters mentioned above and the clinical measurements including the AQ, CAARS, and VFT scores in the ADHD group.

## Discussion

In this study, we applied a unique method of analysis using the cluster-based non-parametric randomization test to compare group differences in the detailed time course of [oxy-Hb] changes in the prefrontal and temporal cortices during the whole baseline and task periods in medication-naïve adults with ADHD and healthy controls. We found that adults with ADHD had significantly smaller [oxy-Hb] activations during the VFT and post-VFT baseline periods in broad prefrontal and temporal areas, relative to those with HC, despite similar VFT performance levels.

We identified that both spatially and temporally connected clusters showing decreased [oxy-Hb] activations in ADHD compared to HC were located in the bilateral PFC (more prominent on the left) and temporal brain regions (more prominent on the left). The brain regions in our findings were consistent with two previous NIRS studies in adults with ADHD. Schecklmann et al. (13) reported that adults with ADHD showed significantly smaller [oxy-Hb] changes during the VFT, compared with HC, in the bilateral inferior frontal gyrus, VLPFC and DLPFC, and parts of the superior temporal gyrus (13). Furthermore, a recent study also revealed similar findings; [oxy-Hb] changes in the left VLPFC and DLPFC during the VFT and post-VFT periods were significantly smaller in adults with ADHD than those recorded in HC (14). The present study, therefore, supports these previous studies, which may further indicate the validity of our findings.

Previous meta-analysis studies using fMRI in HC have indicated that processing of the VFT is associated with the activation of brain regions primarily in the left prefrontal gyrus, particularly in the left VLPFC and DLPFC, and in the medial frontal gyrus (Brodmann area: BA 6, 9, 44, 45, 47) and the anterior cingulate gyrus (BA 24, 32) (31, 32). Moreover, previous neuropsychological studies have reported that deficits in adults with ADHD are widespread, covering multiple cognitive domains including sustained attention and executive functioning (33). Speech fluency is highly correlated with executive functioning and attention capacity, both clinically and experimentally. Specifically, VFT requires effective initiation, cognitive flexibility, and mental shifting skills (34). Therefore, our findings of smaller [oxy-Hb] values during the task period in the prefrontal brain regions could indicate a deficit in brain activations for effective initiation, cognitive flexibility, and mental shifting skills in adults with ADHD.

To our knowledge, this is the first study to compare group differences in the time-courses of [oxy-Hb] changes with a 0.1-s time resolution, since most previous psychiatric NIRS studies average the total values of the [oxy-Hb] changes during the activation task period for each channel and compare the resulting mean values between groups. Thus, when a group difference in [oxy-Hb] changes is confined to a very short time, this conventional averaging method of analysis may miss small differences. However, the cluster-based non-parametric randomization test has the advantage of being able to detect fine differences in [oxy-Hb] activations more clearly. Therefore, this study enabled detailed measurements of time-course changes and provides important insights into differences in executive function related to the VFT between adults with ADHD and HC. For instance, a time-course difference was observed immediately after the activation task in clusters located in the bilateral frontal pole (medial part of inferior and middle frontal gyri). However, this difference only persisted for a short period (about 3–5 s), suggesting that the initial increase in the [oxy-Hb] response to the VFT in the frontal pole is both decreased and slower in adults with ADHD than in HC. On the other hand, hypofrontality in patients with ADHD was seen throughout the task period in the left DLPFC and VLPFC, meaning that despite the fact that the load of the task continued, the initial increase in the left lateral prefrontal hemodynamic response to the task stimulus was smaller and the response failure continued until the end of the task. Briefly, a deficit in the initial activation in the frontal pole and the discontinuance of the left lateral frontal hemodynamic response may reflect the symptoms of ADHD, such as discrepancies in effective initiation, cognitive flexibility, and mental shifting skills. Taken together, our study may provide new neurobiological evidence that prefrontal and temporal [oxy-Hb] activations differ by a certain period of time during the activation task or brain location, possibly representing the pathophysiology of adults with ADHD.

An advantage of this study is that it used participants who had never taken any stimulants or other psychiatric medication at the time of measurement. Therefore, this study excluded the confounding effects of continuous stimulant use on PFC function at the time of the measurement and allowed us to observe unmedicated prefrontal responses in adults with ADHD. Moreover, we confirmed that hypofrontality in ADHD, which may represent a potential biological marker for ADHD, was a consistent finding using a relatively large sample of 101 participants. An advantage of the non-parametric method is the freedom to use any test statistic one considers appropriate. This freedom allows us to solve the MCP in a simple way, and it also allows us to incorporate prior knowledge about the type of effect that can be expected.

**Figure 2:**
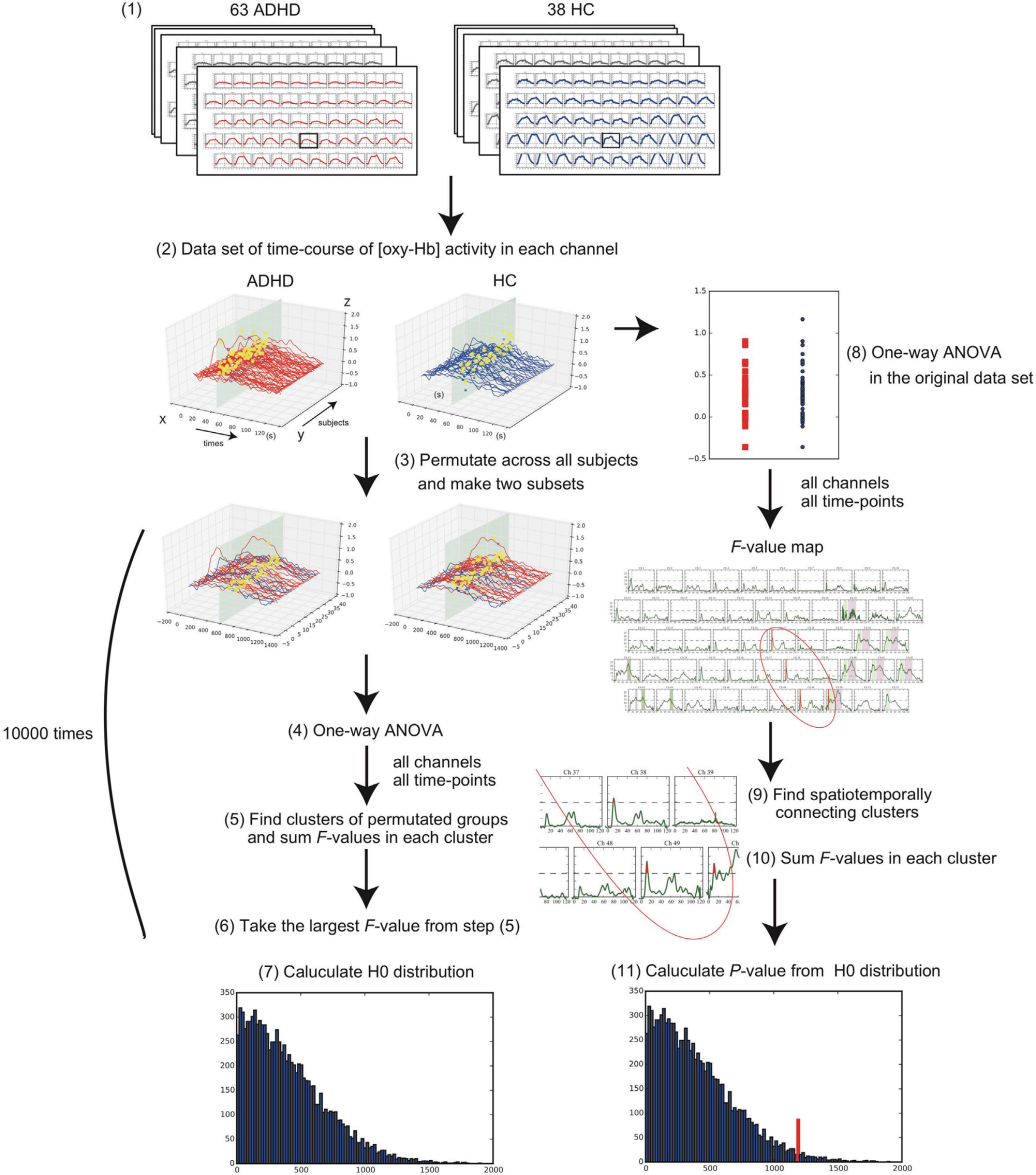
Flowchart of statistical methods for the cluster-based non-parametric randomization test. (1) Collect the 52-channel near-infrared spectroscopy data of the two conditions, consisting of 63 adults with attention-deficit hyperactivity disorder (ADHD) and 38 adults with healthy control (HC). (2) Make a data set of time-course oxygenated-hemoglobin [oxy-Hb] changes for each channel in each condition. Example of one channel is shown. *X*-axis represents time-points; *Y*-axis represents each subject; *Z*-axis represents [oxy-Hb] values. (3) Randomly permutate the data across all the subjects and make two subsets. (4) Perform one-way analysis of variance (ANOVA) and calculate *F* values in all channels and time-points. (5) Find the spatiotemporally connecting clusters where the *F* values are larger than a threshold and calculate the sum of *F* values in each cluster. (6) Take the largest *F*-value from the acquired summed *F* values. (7) Repeat these steps from (3) to (6) 10,000 times and construct a histogram of the largest summed *F* value distribution (see H0 distribution). (8) Perform one-way ANOVA for all channels and time-points in the original data set. (9) Find the spatiotemporally connecting clusters where the *F* values are larger than the threshold. (10) Calculate the sum of the *F* values in each cluster. (11) *P* value is estimated from the summed *F* value of original dataset according to the H0 distribution obtained in step (7).

On the other hand, several limitations of this study should also be noted. First, a cluster-based non-parametric test depends on the threshold that is used to select the samples that will be subsequently clustered. There is no standard way to determine the *F* value threshold required for a cluster-based non-parametric randomization test. Therefore, it is not clear how to choose this threshold to obtain maximum sensitivity for an unknown effect present in the data: for a weak and widespread effect, the threshold should be low, while it should be high for a strong and localized effect. In this study, we plotted the *F* value time series of each channel [Figure 2-(7)]. We found that *F* = 14.9 was the best threshold for acquiring clusters that represented the characteristics of the difference between the two groups [Figure 2-(9); see the dashed line showing the threshold]. Second, since a portion of the HC sample was originally collected for a previous NIRS study examining the effects of antidepressant treatment on brain activation in depression (35), relevant behavioral data, including estimated IQ and AQ scores, were not available. Third, since the AQ scores of the ADHD group were relatively higher in this study, it is possible that this study may have included some adults with autism spectrum disorders (ASD) within the ADHD group; however, at least two psychiatrists had evaluated the ADHD participants and had screened them for ASD comorbidity. Therefore, a future study including HC with relevant behavioral and IQ data is warranted to ensure the consistency of our findings.

In summary, we investigated the hemodynamic changes using the VFT in drug-naïve adult individuals with ADHD and HCs using 52-channel NIRS with a wide coverage over the prefrontal and temporal cortical surface areas. Our study first revealed that brain regions showing abnormal prefrontal and temporal activations in response to the VFT formed spatially and temporally connected clusters in adults with ADHD. We further found that aberrant [oxy-Hb] activation differs according to the time period during the task or according to brain location. These findings suggest that aberrant prefrontal and temporal activation patterns may represent a potential biological marker for ADHD. Considering the safety, low cost, portability, and high temporal resolution of NIRS, the present study provides further evidence to support its potential clinical application in practical psychiatric settings.

## Ethics Statement

The study protocol was approved by the Ethics Committee of Showa University and Tottori University and prepared in accordance to the ethical standards of the Declaration of Helsinki.

## Author Contributions

BY and AI designed the study; BY, SP, and AI performed research; BY, YT, and TI analyzed the data; BY, YT, TI, SP, JH, MM, and AI interpreted the data and wrote the article.

## Conflict of Interest Statement

The authors declare that the research was conducted in the absence of any commercial or financial relationships that could be construed as a potential conflict of interest.
